# Mangiferin Inhibits PDGF-BB-Induced Proliferation and Migration of Rat Vascular Smooth Muscle Cells and Alleviates Neointimal Formation in Mice through the AMPK/Drp1 Axis

**DOI:** 10.1155/2021/3119953

**Published:** 2021-12-03

**Authors:** Qi Wu, Yuanyang Chen, Zhiwei Wang, Xin Cai, Yanjia Che, Sihao Zheng, Shun Yuan, Xiaohan Zhong

**Affiliations:** ^1^Department of Cardiovascular Surgery, Renmin Hospital of Wuhan University, Wuhan, China; ^2^Cardiovascular Surgery Laboratory, Renmin Hospital of Wuhan University, Wuhan, China; ^3^Central Laboratory, Renmin Hospital of Wuhan University, Wuhan, China

## Abstract

Mangiferin is a naturally occurring xanthone C-glycoside that is widely found in various plants. Previous studies have reported that mangiferin inhibits tumor cell proliferation and migration. Excessive proliferation and migration of vascular smooth muscle cells (SMCs) is associated with neointimal hyperplasia in coronary arteries. However, the role and mechanism of mangiferin action in neointimal hyperplasia is still unknown. In this study, a mouse carotid artery ligation model was established, and primary rat smooth muscle cells were isolated and used for mechanistic assays. We found that mangiferin alleviated neointimal hyperplasia, inhibited proliferation and migration of SMCs, and promoted platelets derive growth factors-BB- (PDGF-BB-) induced contractile phenotype in SMCs. Moreover, mangiferin attenuated neointimal formation by inhibiting mitochondrial fission through the AMPK/Drp1 signaling pathway. These findings suggest that mangiferin has the potential to maintain vascular homeostasis and inhibit neointimal hyperplasia.

## 1. Introduction

Coronary artery disease (CAD) is a growing health burden worldwide [1]. Neointimal hyperplasia of the coronary arteries is the major pathophysiological change associated with CAD [2]. Several therapeutic procedures such as balloon angioplasty, rotational atherectomy, percutaneous coronary intervention, and coronary artery bypass grafting (CABG) are used in the treatment of CAD. However, the development of restenosis caused by mechanical injuries to the coronary arteries is the most common complication, which is associated with poor prognosis and decreased long-term survival of patients with CAD [3]. The transformation of the phenotype of SMCs plays an important role in neointimal formation [4]. Current therapeutic strategies against neointimal hyperplasia are not effective for patients with CAD. Therefore, there is urgent need to identify and develop novel therapeutic agents for the treatment of coronary artery restenosis.

Mitochondria are known to produce the majority of ATP necessary for normal cellular function [5]. Relevant studies have revealed that the mitochondria are highly dynamic organelles that continually undergo fission and fusion events in order to maintain their size, distribution, and shape [5]. This process is referred to as mitochondrial dynamics. Moreover, alternations in mitochondrial function greatly influence cell fate [6]. Recent evidence supports the correlation between mitochondrial dynamics and vascular disease [7]. Mfn1 and Mfn2 are proteins located in the outer membrane of the mitochondria that are mainly involved in mitochondrial fusion [8]. Inhibition of mitochondria fission by suppressing translation of Mfn1 induces senescence of SMCs in vessels [9]. Moreover, miR-93 promotes proliferation, migration, and neointimal information of SMCs by inhibiting Mfn2 expression [10]. Drp1 plays a central role in mitochondria fission. A decrease in phosphorylation of Drp1 at Ser 616 significantly inhibits the proliferation, migration, and neointimal formation of SMCs [11]. Therefore, mitochondrial dynamics is critical for progression of neointimal hyperplasia.

AMP-activated protein kinase (AMPK) is extremely important in the regulation of energy metabolism in cells. It is a heterotrimeric complex consisting of a catalytic *α*-subunit and two regulatory subunits, *β* and *γ* [12]. Usually, AMPK is mainly activated by a decrease in ATP levels, with mitochondria being the main source of ATP [13]. Recent research findings revealed that AMPK is closely associated with mitochondrial dynamics and that AMPK facilitates mitochondrial fusion by regulating proteins involved in mitochondrial dynamics [14]. This indicates that AMPK is a promising regulator of mitochondrial function.

Mangiferin was first extracted from a mango tree and is mainly composed of polyphenolic C-glucosyl-xanthone. Mangiferin has previously been shown to have antioxidant, anti-inflammatory, and antitumor activities [15–18]. It has also been associated with the progression of various diseases by modulating mitochondrial function [19–21]. Intriguingly, mangiferin was also identified as a potent AMPK activator [22]. Treatment with magniferin resulted in enhanced mitochondrial bioenergetics, increased mitochondrial membrane potential, inhibition of caspase-3 activation, and downregulation of cytochrome C leakage [22]. Accordingly, the involvement of mangiferin in the regulation of mitochondrial function via the activated AMPK signal pathway was shown. However, there are very few reports on the role of magniferin in the regulation of mitochondrial dynamics in SMCs and the maintenance of the normal structure and function of mitochondria in vessels. Since there are a high number of patients with CAD, there is a need to determine the specific mechanism by which mangiferin attenuates neointimal formation.

In this study, we demonstrated that mangiferin protects the arteries against neointima hyperplasia. Mangiferin exerted this effect by decreasing the phosphorylation of Drp1 at Ser-616 and promoting mitochondrial fusion through AMPK activation. In line with this finding, we found that the inhibition of AMPK by compound C (CC) reversed the mangiferin-induced inhibition of neointima hyperplasia. Thus, the positive effect of mangiferin is AMPK/Drp1 dependent. These findings indicate for the first time that mangiferin attenuates neointimal hyperplasia through the AMPK/Drp1 axis and that mangiferin is a potential natural compound for the treatment of restenosis artery disease.

## 2. Materials and Methods

### 2.1. Compounds and Reagents

Mangiferin was purchased from Yuanye Bio-Technology Co., Ltd. (Shanghai, China) and dissolved in DMSO. TRIzol was obtained from Yeasen Biotech Co., Ltd. (Shanghai, China). The First-Strand cDNA Synthesis Kit and SYBR Green qPCR Master Mix were purchased from Servicebio Biotech Co., Ltd. (Wuhan, China). MitoTracker Red CM-H2XRos was purchased from Yeasen Biotech Co., Ltd. (Shanghai, China), while compound C (CC) was purchased from MedChemExpress (New Jersey, USA). The Anti-AMPK, Anti-Phospho-AMPK*α* (Thr172), Anti-Drp1, and Anti-Phospho-DRP1 (Ser616) antibodies were obtained from Affinity Biosciences LTD (New Jersey, USA), while Anti-MMP2, Anti-SM22*α*, Anti-*α*-SMA, Anti-PCNA, Anti-CNN1, and Anti-*β*-Actin were purchased from Servicebio Biotech Co., Ltd. (Wuhan, China). Anti-Mfn1, Anti-Mfn2, Anti-Fis1, and Anti-OPA1 antibodies were purchased from Beyotime Biotechnology Co., Ltd. (Shanghai, China).

### 2.2. Isolation and Identification of Primary Rat Aortic Smooth Muscle Cells

The animal protocols used in this study were approved by the Animal Research Ethics Committee of Wuhan University. The rats were obtained from Hubei province center for disease control and prevention and weighed between 130 g and approximately 150 g. Pentobarbitone was used to anesthetize the rats, followed by isolation of the aortic arteries under sterile conditions. Fresh isolated aortas were irrigated thrice with PBS, stripping adventitia and intima in PBS with microinstruments. The aorta tissues were then cut into pieces of 2 mm^2^, attached to 60 mm cell culture dishes, and incubated for 30 minutes. Thereafter, fresh DMEM/F12 medium supplemented with 20% FBS and 1% penicillin streptomycin solution was added, and floating aorta tissues were removed. The medium was replaced three days later. The tissues were incubated until SMCs crawled out of the aorta tissue to form a monolayer (the cell confluency was 50%) on the dish. The aorta tissues were then removed, the cells washed thrice with PBS and digested with 0.5% pancreatin. Digestion was terminated by adding the growth medium. The SMCs were collected and planted in 60 mm dish. Primary rat aortic smooth muscle cells in the third passage were used in this study.

### 2.3. RT-PCR

100 ng total RNA was reverse transcribed to cDNA and used for RT-PCR. RT-PCR was carried out as previously described [23] to detect the mRNA expression levels of PCNA and MMP2. The PCR primer sequences used in this study are shown in Table 1.

### 2.4. Western Blot

For western blot, 20 *μ*g total protein was loaded into each lane of SDS-PAGE gels. The specific protocol has been previously described [23]. The anti-mouse IgG (H+L) (DyLight™ 800 4x PEG Conjugate) and anti-rabbit IgG (H+L) (DyLight™ 800 4x PEG Conjugate) secondary antibodies were obtained from Cell Signaling Technology (Boston, US). All the bands were visualized using Odessy fluorescence imaging system (Li-Cor). The primary antibodies and their dilutions as used in this study are shown in Table 2.

### 2.5. Cell Migration Assay

Transwell chambers (8 mm pore size; Corning) were used to analyze the migration ability of the SMCs. Briefly, SMCs were serum starved for 24 h, and then, 1 × 10^4^ SMCs were seeded in the upper chambers with or without mangiferin. DMEM/F12 medium supplemented with 10% FBS with or without PDGF-BB was added to the lower chamber. The cells were incubated at 37°C for 9 hours in a CO_2_ incubator. Thereafter, the medium was removed and the cells washed thrice with PBS. The upper chambers were then fixed with 4% paraformaldehyde for 15 minutes at room temperature. SMCs in the upper layer were gently wiped off using a cotton swab. The fixed cells were stained using 0.2% crystal violet stain for 20 minutes at room temperature, and the excess crystal violet washed with water. Cells were photographed under a microscope, and ImageJ was used to quantify the number of cells in different fields of view.

### 2.6. Flow Cytometry

RSMCs were seeded in 60 mm cell culture dishes and starved for 24 hours for synchronization. The cells were then pretreated with mangiferin for 6 hours and then treated with 20 ng/ml PDGF-BB for 24 hours. Thereafter, RSMCs were collected using trypsin digestion. Dihydroethidium (DHE) at final concentration of 10 *μ*M was used to label the ROS in RSMCs for 60 min at 37°C, followed by detection using flow cytometry (excitation wavelength 488 nm, emission wavelength 575 nm).

### 2.7. Animal

Animal experiments were designed strictly in accordance with the Care and Use of Laboratory Animals published by the National Institutes of Health and approved by the ethical committee of the Renmin Hospital of Wuhan University (approval no.: WDRM20201107). Animals were obtained from Hubei provincial Center for Disease Control and Prevention, fed a standard diet and had free access to water. Mice were maintained in 12 hour light/dark cycles at 22°C.

Carotid ligations were performed in 12-week-old male C57BL/6J mice. Briefly, mice were first anesthetized using sodium pentobarbital. A middle neck incision was made, followed by the exposure and separation of the left common carotid artery, which was then ligated with 6-0 silk. Twelve C57BL/6J mice were divided randomly into two groups. Before ligation, one group was administered with intraperitoneal injections of mangiferin, while the other group was administered with intraperitoneal injections of corn oil for one week. After surgery, mangiferin was administered once daily for four weeks. Thereafter, the mice were anesthetized using pentobarbital sodium, followed by harvesting of the left common carotid arteries. The right common carotid arteries were also harvested and served as the controls. Finally, mice were sacrificed using high doses of pentobarbital sodium.

### 2.8. Histopathology Analysis

The right and left carotid arteries were collected and washed with precooled saline to remove excess blood. All collected tissues were fixed using 4% paraformaldehyde solution for 24 hours. The samples were then dehydrated in gradient concentration of alcohol and transparency with xylene, embedded in paraffin, and sectioned. Afterwards, the sections were dewaxed using xylene and hydrated in gradient alcohol. The size of intima and media was determined by staining the sections using Elastica Van Gieson (EVG) staining solution.

### 2.9. Immunofluorescence

Cells were cultured at a density of 1 × 10^5^ cells per well in six-well plates containing a cover slip. After 24 hours, RSMCs were serum starved for 24 hours for synchronization. The RSMCs were then pretreated with mangiferin or equal volumes of dimethyl sulfoxide (DMSO), followed by treatment with or without 20 ng/ml PDGF-BB for 24 hours. Thereafter, the RSMCs were washed with PBS to remove excess culture medium and fixed with 4% paraformaldehyde for 15 minutes. The RSMCs were permeabilized using 0.2% Triton solution and blocked with goat serum for 60 minutes. The cells were then incubated in primary antibody at 4°C overnight, followed by incubation in Cy3 labeled secondary antibody for 1 hour at room temperature, and finally observed under a fluorescence microscope.

### 2.10. Mitochondrial Staining

Cells were seeded in six-well plates at a density of 1 × 10^5^ cells per well. The cells were then treated, and thereafter, the media was removed, and the cells were stained with MitoTracker Red CM-H2XRos at a final concentration of 125 nM for 30 minutes at 37°C (excitation wavelength 579 nm, emission wavelength 599 nm). Afterwards, the staining solution was discarded, and the RSMCs washed with PBS thrice. Finally, the cells were observed under a fluorescence microscope and ImageJ software was used to analyze mitochondrial morphology.

### 2.11. Statistical Analysis

All the assays in this study were carried out in triplicates, and the data were expressed as mean ± SD. Unpaired *t*-test or Mann–Whitney *U* tests were used to compare the means between two groups, while one-way ANOVA was used to compare the means among three or more groups. A *P* < 0.05 was considered to be statistically significance. GraphPad Prism 7.0 was used for statistical analysis.

## 3. Result

### 3.1. Mangiferin Inhibited PDGF-BB-Induced Proliferation and Migration of RSMCs

**Figure 1:**
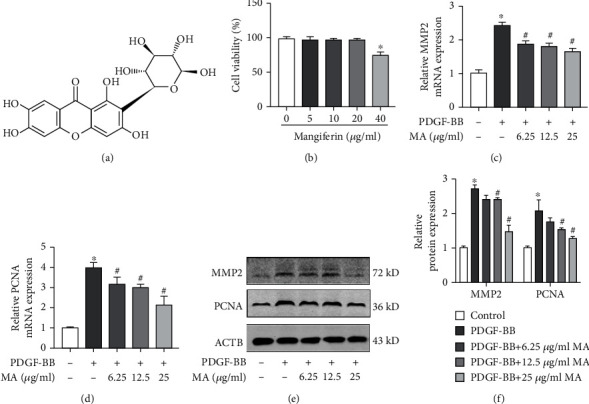
Mangiferin inhibited PDGF-induced proliferation and migration of RSMCs. (a) Chemical structure of mangiferin. (b) The viability of RSMCs was determined using CCK-8 assay after treatment with different concentrations of mangiferin for 24 hours. (c, d) The mRNA expression levels of PCNA and MMP2 as measured using RT-PCR. (e, f) The protein expression levels of PCNA and MMP2 as determined using western blot. All assays were conducted in triplicates and data presented as mean ± S.D. ^∗^*P* < 0.05, compared with the control group. ^#^*P* < 0.05, compared with the PDGF-BB group.

**Figure 2:**
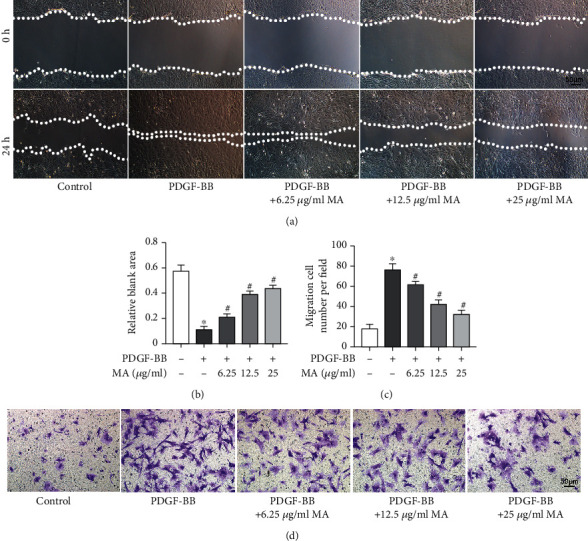
Mangiferin inhibited the PDGF-BB-induced proliferation and migration of RSMCs. (a, b) The migration ability of RSMCs was evaluated using the scratch assay after treatment with various concentrations of mangiferin with or without 20 ng/ml PDGF-BB for 24 hours. (c, d) The migration ability of RSMCs was assessed using Transwell assay after treatment with different concentrations of mangiferin with or without 20 ng/ml PDGF-BB for 24 hours. All assays were conducted in triplicates and data presented as mean ± S.D. ^∗^*P* < 0.05, compared with the control group. ^#^*P* < 0.05, compared with the PDGF-BB group.

The chemical structure of mangiferin is shown in Figure 1(a). The effect of mangiferin on cell viability was determined using the CCK-8 assay, with the results showing that 50 *μ*g/ml mangiferin significantly inhibited the survival of RSMCs. However, mangiferin at concentrations between 6.25 and approximately 25 *μ*g/ml did not affect cell survival (Figure 1(b)). Consequently, concentrations of mangiferin below 25 *μ*g/ml were not used in subsequent assays. Cell proliferation and migration have been implicated in the pathophysiology of artery restenosis [24]. Mangiferin decreased the PDGF-BB-induced expression of MMP2, which is associated with cell migration (Figure 1(c)). In addition, magniferin decreased the mRNA levels of PCNA, which is associated with cell proliferation capacity (Figure 1(d)). The results of western blot were consistent with the results of qPCR (Figures 1(e) and 1(f)). The results of Transwell migration assay and scratch assay indicated that mangiferin significantly inhibited PDGF-BB-induced migration of RSMCs (Figures 2(a)–2(d)).

### 3.2. Mangiferin Inhibits PDGF-BB-Induced Dedifferentiation of RSMCs

**Figure 3:**
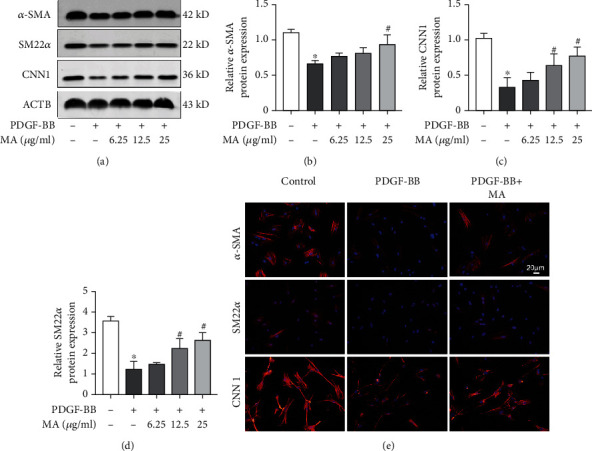
Mangiferin inhibited PDGF-BB-induced dedifferentiation of RSMCs. (a–d) The protein expression levels of *α*-SMA, SM22*α*, and CNN1 were detected using western blot after RSMCs were treated with different concentrations of mangiferin with or without 20 ng/ml PDGF-BB for 24 hours. (e) The expression levels of *α*-SMA, SM22*α*, and CNN1 were determined using immunofluorescence after RSMCs were treated with mangiferin with or without 20 ng/ml PDGF-BB for 24 hours. All assays were conducted in triplicates and all data presented as mean ± S.D. ^∗^*P* < 0.05, compared with the control group. ^#^*P* < 0.05, compared with the PDGF-BB group.

Phenotypic transformation is a common event in the pathophysiology of vascular disease [25].*α*-SMA, SM22*α*, and CNN1 are the markers of the contractile SMCs. To investigate the effect of mangiferin on changes in RSMCs phenotype, we detected the protein expression levels of *α*-SMA, SM22*α*, and CNN1 using western blot. PDGF-BB induced a significant decrease in the expression levels of *α*-SMA, SM22*α*, and CNN1, but mangiferin was able to reverse this effect in a dose-dependent manner (Figures 3(a)–3(d)). The results of immunofluorescence were consistent with those of western blot (Figure 3(e)).

### 3.3. Mangiferin Alleviated PDGF-BB-Induced Mitochondrial Fission and Increase in ROS Levels

**Figure 4:**
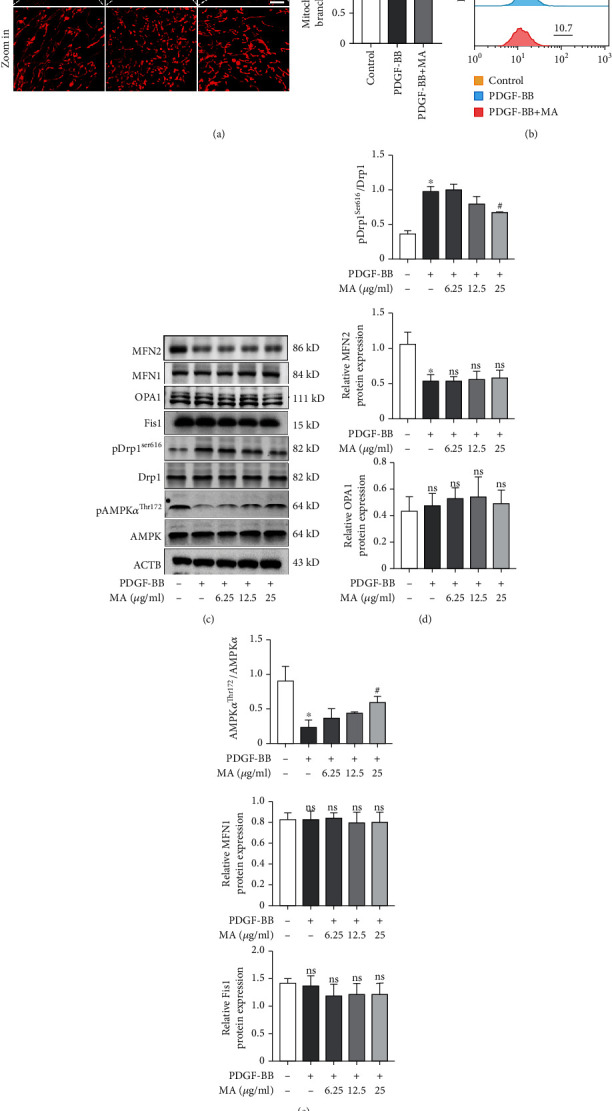
Mangiferin alleviated PDGF-BB-induced mitochondrial fission and increase in ROS levels. (a) RSMCs were stained with mitotracker probe after treatment with different concentrations of mangiferin with or without 20 ng/ml PDGF-BB for 24 hours. The average length of mitochondria was quantified using ImageJ software. (b) Intracellular ROS levels were detected using flow cytometry after RSMCs were treated with mangiferin with or without 20 ng/ml PDGF-BB for 24 hours. (c–e) Western blot was used to measure the protein expression levels of AMPK, pAMPKThr172, Drp1, and pDrp1Ser616 after RSMCs were treated with various concentrations of mangiferin with or without 20 ng/ml PDGF-BB for 24 hours. All assays were conducted in triplicate and all data presented as mean ± S.D. ^∗^*P* < 0.05, compared with the control group. ^#^*P* < 0.05, compared with the PDGF-BB group.

Mitochondrial fission and fusion are closely associated with neointimal formation and the functional status of RSMCs [26]. Findings from a previous study indicated that PDGF-BB stimulated the proliferation of RSMCs partly by promoting mitochondrial fission. Staining of mitochondrial RSMCs with mitotracker staining solution demonstrated that PDGF-BB promoted mitochondrial fission and mangiferin partly reversed this effect (Figure 4(a)). Reactive oxygen species (ROS) are mainly produced during oxidative phosphorylation in the mitochondria and are closely associated with mitochondrial function. Results of flow cytometry showed that mangiferin significantly decreased PDGF-BB-induced production of ROS, further indicating its role in modulating mitochondrial function (Figure 4(b)). Mitochondrial dynamics are highly associated with the expression of mitochondrial fusion-fission-related proteins and AMPK phosphorylation. We therefore evaluated the expression of mitochondrial fusion-fission-related proteins after treatment with or without PDGF-BB and mangiferin using western blot. PDGF-BB dramatically promoted the phosphorylation of Drp1 at Ser-616 (Figures 4(c) and 4(d)), while mangiferin partly reversed this effect in a dose-dependent manner. In addition, mangiferin also increased the expression of AMPK at Thr172 (Figures 4(c) and 4(e)). Moreover, treatment with PDGF-BB significantly decreased the expression of Mfn2 but did not significantly alter the protein levels of Mfn1, OPA1, and Fis1. However, mangiferin failed to reverse this effect.

### 3.4. Mangiferin Significantly Suppressed PDGF-BB-Induced RSMC Phenotype Transformation through the AMPK/Drp1 Axis

**Figure 5:**
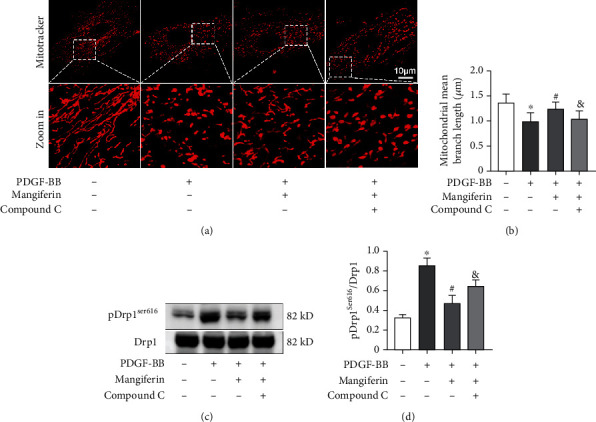
Mangiferin significantly suppressed PDGF-BB-induced RSMC phenotype transformation through the AMPK/Drp1 axis. (a, b) AMPK inhibitor, compound C, reverses the inhibitory effects of mangiferin on PDGF-BB-induced mitchondrial fission. (c, d) The protein expression levels of Drp1, and pDrp1^Ser616^ was determined using western blot after RSMCs were treated with or without PDGF-BB, mangiferin, and compound C.

Mitochondria are the main organelles involved in energy metabolism, while AMPK plays a vital role in monitoring the energy metabolism status of cells and mitochondria [14, 27]. A previous study reported that mangiferin activates the AMPK signaling pathway [28]. This was consistent with findings from our study which showed that mangiferin increased AMPK phosphorylation in RSMCs treated with PDGF-BB. To investigate the underlying mechanism, activation of AMPK was inhibited using compound C (CC), which is a specific inhibitor of AMPK [29]. This eliminated the mangiferin-induced inhibition of mitochondrial fission (Figures 5(a) and 5(b)). In addition, CC significantly downregulated the phosphorylation of Drp1 at Ser-616 after treatment with mangiferin (Figures 5(c) and 5(d)). Therefore, the inhibition of PDGF-BB-induced RSMC proliferation, migration, and phenotype change by mangiferin may be mediated through the AMPK/Drp1 signaling pathway.

### 3.5. Mangiferin Attenuated Neointimal Formation In Vivo

**Figure 6:**
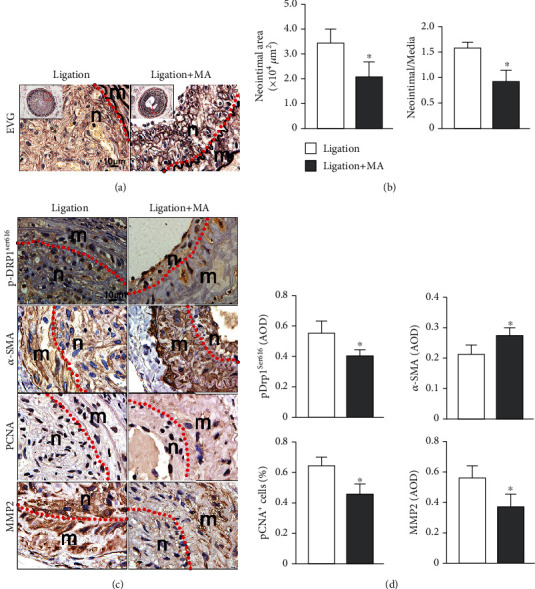
Mangiferin attenuated neointimal formation in vivo. (a) Intimal area and the ratio of neointima/media were measured after carotid ligation treated with or without mangiferin (*n* = 6). (b) pDrp1Ser616, *α*-SMA, PCNA, and MMP2 expression levels were detected using immunohistochemistry in ligated carotid arteries treated with or without mangiferin. All data were presented as mean ± S.D. ^∗^*P* < 0.05, compared with the control group. ^#^*P* < 0.05, compared with the PDGF-BB group.

To further verify the effect of mangiferin on neointimal hyperplasia, the mouse carotid artery ligation model was established. EVG staining was used to measure the size of the intima and media of carotid artery (Figure 6(a)). In this mouse model, our results showed that mangiferin dramatically decreased the ratio of intima/media compared with the ligation group (Figure 6(b)). Moreover, mangiferin decreased p-Drp1ser-616 expression and inhibited neointimal formation. In addition, there was downregulation of PCNA and MMP2, as well as upregulation of *α*-SMA in mice treated with mangiferin compared to the ligation group (Figures 6(c) and 6(d)). These results indicated that mangiferin inhibited neointimal hyperplasia by modulating the AMPK/Drp1 signaling pathway.

## 4. Discussion

Mangiferin is a natural compound with multiple positive effects on various diseases [30]. Our results indicate that mangiferin reverses the PDGF-BB-induced proliferation, migration, and phenotype change of RSMCs. In addition, mangiferin inhibited PDGF-BB-induced upregulation of Drp1 expression in RSMCs and induced phosphorylation of AMPK. Since AMPK has been shown to modulate mitochondrial dynamics, we postulated that mangiferin prevents neointimal hyperplaisa by modulating the AMPK/Drp1 signaling pathway. As expected, treatment with PDGF-BB and mangiferin inhibited the activation of AMPK in RSMCs and induced pDrp1Ser616 upregulation. Moreover, mangiferin significantly inhibited neointimal hyperplasia in the mouse carotid artery ligation model. Overall, our data strongly suggested that mangiferin inhibited neointimal formation in vitro and in vivo through the AMPK/Drp1 signaling pathway.

AMPK plays a vital role in modulating cellular energy balance and is activated when the ratio of AMP/ATP or ADP/ATP increases. It is well established that AMPK has potent anti-inflammatory, antioxidant, and antitumor effects [31, 32]. Several studies have demonstrated that AMPK plays a protective role in various vascular diseases [33, 34]. Mangiferin has been reported to regulate hepatic lipid metabolism via AMPK activation [35]. Therefore, we postulated that mangiferin reversed PDGF-BB-induced effects on RSMCs by activating AMPK. Metformin modulates SMC phenotype transformation by activating AMPK, thus inhibiting intracranial aneurysm progression [36]. In our study, we found that mangiferin indeed induced AMPK phosphorylation in RSMCs treated with PDGF-BB possibly through multiple mechanisms and that AMPK plays a central role in the modulation of RSMC function by mangiferin. On the one hand, mangiferin has been shown to promote glycolysis and increase the production of alpha-ketoglutarate, thus promoting AMPK phosphorylation [19]. On the other hand, ROS, which is mainly produced during mitochondrial oxidative phosphorylation, also plays a vital role in normal physiological processes [37]. However, excessive ROS accumulation also leads to mitochondrial dysfunction [38]. In this study, we found that PDGF-BB promoted generation and accumulation of ROS in RSMCs. The accumulation of ROS inhibited oxidative phosphorylation leading to decreased production of ATP, increased ADP/ATP or AMP/ATP ratio, which in turn activated the AMPK signaling pathway.

Mitochondrial dynamics plays a significant role in modulating neointimal formation, and Fis1, Drp1, OPA1, Mfn1, and Mfn2 have been closely associated with mitochondrial fission and fusion [39]. Preventing mitochondrial fission can inhibit neointimal hyperplasia as well as the proliferation and migration of SMCs [11]. PDGF-BB stimulated mitochondrial fission by increasing expression of pDrp1 (Ser616) in SMCs [40]. In our study, we found that mangiferin decreased the protein levels of pDrp1 (Ser616) in RSMCs. Drp1 is mainly located in cytoplasm, but phosphorylation at serine 616 promotes it recruitment to the outermembrane of mitochondria and accelerates mitochondrial fission. In contrast, phosphorylation of Drp1 at serine 637 has opposite effects. In this study, mangiferin downregulated the phosphorylation of Drp1 at serine 616. ROS plays a dual role in the modulation of physiological functions of the cell. Moderate levels of ROS promote proliferation, migration, and dedifferentiation of SMCs, while high levels of ROS impair mitochondrial function, leading to a decrease in ATP synthesis, and inducing cell apoptosis [41].

Drp1 activity is strictly regulated by AMPK in SMCs, and different roles of AMPK in modulating Drp1 have been illustrated [42–44]. The activation of AMPK alleviated lead-induced phosphorylation of Drp1 (Ser616) [45]. However, another study reported that the activation of AMPK induced mitochondrial biogenesis by increasing phoshorylation of Drp1 (Ser616) to attenuate carbon tetrachloride-induced liver fibrosis in rats [46]. Several factors may account for these inconsistencies. Mitochondrial fission accelerates the rates of oxidative phosphorylation to some extent, thus increasing the levels of ATP in vitro or in vivo [44]. Since AMPK acts as an energy sensor in cells, it may promote mitochondrial fission by inducing Drp1 phosphorylation at serine 616 when ATP levels decline in vivo or in vitro. However, excessive mitochondrial fission leads to mitochondrial fragmentation, which triggers mitochondrial autophagy caused by mitochondrial dysfunction. Ultimately, the production of ATP is reduced, the AMPK signaling pathway is activated; AMPK downregulated Drp1 and contributed to mitochondrial fusion, restored mitochondrial function, and accelerated ATP production [47]. In our study, inhibition of AMPK activation by CC significantly decreased the phosphorylation of Drp1 at serine 616 and enhanced mitochondrial division in RSMCs. Taken together, mangiferin inhibited the proliferation, migration, and dedifferentiation of RSMCs mainly through the AMPK/Drp1 signaling pathway, ultimately attenuating neointimal formation.

There are limitations to our study. All assays were only repeated three times, yet repeating them five times would have increased the reliability of the results. Since several proteins are involved in modulating mitochondrial fusion and fission, there may be multiple mechanisms involved in the inhibition of neointimal formation by mangiferin other than our proposed mechanism. Therefore, there is need to explore possible alternative mechanisms. Moreover, in this study, 10 *μ*M DHE were used to detect the ROS levels in RSMCs, the high concentrations of DHE could cause cellular membrane depolarization, this might have leaded to slightly bias in the current data, and improvement will be made in our next work.

**Figure 7:**
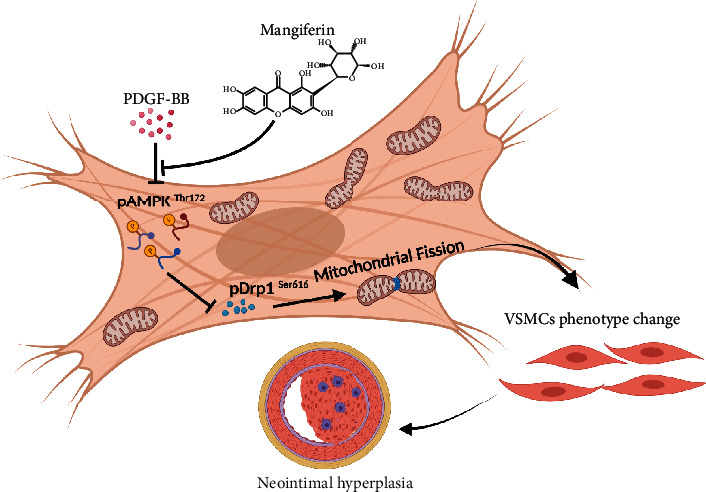
Diagram of mangiferin restraining neointimal hyperplasia. In the normal condition, VSMCs maintain contraction type, which are crucial for maintaining the physiology function of the coronary artery. PDGF-BB potent induces cell proliferation, migration, and phenotype change of VSMCs during neointimal hyperplasia. In this study, PDGF-BB inhibited AMPK phosphorylation activation at Thr 172, Drp1 was phosphorylation activated at Ser616, and then, Drp1 recruits to mitochondria accelerating mitochondrial fission, subsequently, VSMCs in coronary artery transform from contraction type to synthesis type, ultimately, contributing to the neointimal formation. However, we found that mangiferin effectively reversed the effect of PDGF-BB-induced neointimal formation.

In summary, mangiferin significantly inhibited neointimal hyperplasia induced by mouse carotid ligation by inhibiting proliferation, migration, and dedifferentiation of RSMCs. Mangiferin also inhibited PDGF-BB-induced mitochondrial fission, increased the phosphoration levels of AMPK, and significantly downregulated Drp1. Meanwhile, Drp1 is tightly modulated by AMPK. Mechanistically, AMPK inhibition reversed the mangiferin-induced positive effects on RSMCs and blocked the mangiferin-induced downregulation of Drp1 phosphorylation at serine 616; the specific mechanism is presented in Figure 7. Our results indicate that AMPK acts upstream of Drp1, inhibits Drp1 recruitment to the mitochondrial, and inhibits mitochondrial division. Taken together, mangiferin significantly ameliorated neointimal hyperplasia by modulating the AMPK/Drp1 signaling pathway.

## Figures and Tables

**Table 1:** qRT-PCR primer sequences.

Gene	Primer sequence
PCNA	Forward primer, 5′-TCCGAAGGCTTCGACACATAC-3′
	Reverse, 5′-GGACATGCTGGTGAGGTTCA-3′
MMP2	Forward, 5′-ACCTTGACCAGAACACCATCGAG-3′
	Reverse, 5′-CAGGGTCCAGGTCAGGTGTGTA-3′

**Table 2:** Primary antibody information and dilution in this research.

Antibody	Company	Catalog	Dilution		
			WB	IHC	IF
MMP2	Servicebio	GB11130	1 : 1000	1 : 200	—
PCNA	Servicebio	GB11010	1 : 2000	1 : 300	—
ACTB	Servicebio	GB11001	1 : 3000	—	—
*α*-SMA	Servicebio	GB111364	1 : 1000	1 : 500	1 : 200
SM22*α*	Servicebio	GB11366	1 : 1000	—	1 : 100
CNN1	Servicebio	GB11954	1 : 1000	—	1 : 500
MFN1	Beyotime	AF7461	1 : 1000	—	—
MFN2	Beyotime	AF7473	1 : 1000	—	—
OPA1	Beyotime	AF7653	1 : 1000	—	—
Fis1	Beyotime	AF8268	1 : 1000	—	—
Drp1	Beyotime	AF6735	1 : 1000	—	—
Drp1 ser616	Affinity	AF8470	1 : 1000	1 : 100	—
AMPK	CST	#2532	1 : 1000	—	—
P-AMPK	CST	#2535	1 : 1000	—	—

## Data Availability

The data used to support the findings of this study are available from the corresponding author upon request.
